# Origins of Giant Dielectric Properties with Low Loss Tangent in Rutile (Mg_1/3_Ta_2/3_)_0.01_Ti_0.99_O_2_ Ceramic

**DOI:** 10.3390/molecules26226952

**Published:** 2021-11-17

**Authors:** Nateeporn Thongyong, Narong Chanlek, Pornjuk Srepusharawoot, Prasit Thongbai

**Affiliations:** 1 Giant Dielectric and Computational Design Research Group (GD–CDR), Department of Physics, Faculty of Science, Khon Kaen University, Khon Kaen 40002, Thailand; th.nateeporn@kkumail.com (N.T.); spornj@kku.ac.th (P.S.); 2 Synchrotron Light Research Institute (Public Organization), 111 University Avenue, Muang District, Nakhon Ratchasima 30000, Thailand; narong@slri.or.th

**Keywords:** TiO_2_, giant/colossal permittivity, XPS, dielectric relaxation, X9R capacitor

## Abstract

The Mg^2+^/Ta^5+^ codoped rutile TiO_2_ ceramic with a nominal composition (Mg_1/3_Ta_2/3_)_0.01_Ti_0.99_O_2_ was synthesized using a conventional solid-state reaction method and sintered at 1400 °C for 2 h. The pure phase of the rutile TiO_2_ structure with a highly dense microstructure was obtained. A high dielectric permittivity (2.9 × 10^4^ at 10^3^ Hz) with a low loss tangent (<0.025) was achieved in the as-sintered ceramic. After removing the outer surface, the dielectric permittivity of the polished ceramic increased from 2.9 × 10^4^ to 6.0 × 10^4^, while the loss tangent also increased (~0.11). The dielectric permittivity and loss tangent could be recovered to the initial value of the as-sintered ceramic by annealing the polished ceramic in air. Notably, in the temperature range of −60–200 °C, the dielectric permittivity (10^3^ Hz) of the annealed ceramic was slightly dependent (<±4.4%), while the loss tangent was very low (0.015–0.036). The giant dielectric properties were likely contributed by the insulating grain boundaries and insulative surface layer effects.

## 1. Introduction

Multilayer ceramic capacitors (MLCCs) are essential components in microelectronics applications throughout the development of technology. With the rapid growth of the electronic industry, the efficiency potential of MLCCs is demanded. The crucial way to significantly improve the properties of MLCCS is to use a giant dielectric material with a high dielectric permittivity of ε′ > 10^4^. Numerous types of giant dielectric material have been developed in past decades, such as CaCu_3_Ti_4_O_12_ (CCTO) and related ACu_3_Ti_4_O_12_ compounds [1,2,3,4,5,6], CuO [7], ***A***Fe_1/2_***B***_1/2_O_3_ (***A*** = Ba, Sr, Ca; ***B*** = Nb, Ta, Sb) [8], (Li, Ti) doped NiO [9], and codoped SrTiO_3_-based ceramics [10]. However, the dielectric loss tangent (tanδ) of most giant dielectric oxides is still huge (>>0.1), which is not required for use in MLCCs. Thus, the importance of developing MLCCs with good temperature stability and electroceramics for high energy density capacitors has been widely reported [11,12,13].

In 2013, the first experiment on TiO_2_ codoped with acceptors/donors was reported [14]. The (ln_1/2_Nb_1/2_)_0.1_Ti_0.90_O_2_ exhibited outstanding dielectric performance properties, including a high ε′ (>10^4^), low tanδ (<0.02), and slight temperature dependence of ε′ over a wide range. A new approach to this research is the electron-pinned defect dipole (EPDDs) mechanism, which was proposed as the source of the excellent dielectric behavior. Free electrons in the rutile TiO_2_ structure, which can be produced by doping with Nb^5+^, are localized in the $2ln^{3+}+V_{o}^{••}+2Ti^{3+}+2Nb^{5+}+Ti^{4+}$ defect clusters under an applied electric field. Codoping an electron donor into the rutile TiO_2_ structure can produce a high ε′ with a low tanδ. The substitution of TiO_2_ with electron acceptor dopants was necessary to reduce tanδ by restricting the long range movement within defect clusters.

The enhancement of the giant dielectric properties of TiO_2_ by codoping it with acceptor–donor dopants has been intensively studied, including Ga^3+^/Nb^5+^ [15], Ga^3+^/Ta^5+^ [16], Al^3+^/Nb^5+^ [17], Al^3+^/Ta^5+^ [18], Zn^2+^/Nb^5+^ [19,20], and Ag^+^/Nb^5+^ (or Ta^5+^/W^6+^) [21,22,23]. Wei et al. [24] reported significantly enhanced dielectric properties with ε′ ≈ 3.0 × 10^4^ and low tanδ ≈ 0.05. Dong et al. [24] reported a significant improvement of the dielectric properties of Mg^2+^/Ta^5+^ codoped TiO_2_ ceramics, which were described by the formation of the EPDDs. According to the previous works, the internal barrier layer capacitor (IBLC) [25,26,27], surface barrier layer capacitor (SBLC) [15,28,29], and small polaron hopping models [30,31] were also proposed to be the primary origins of the giant dielectric properties of the Ga^3+^/Ta^5+^, Al^3+^/Ta^5+^, and In^3+^/Nb^5+^ codoped TiO_2_ ceramics. Furthermore, broad-band dielectric spectroscopy up to the THz IR range showed that the giant dielectric response in codoped TiO_2_ ceramics was clearly attributed to the IBLC effect [32,33]. Therefore, it is important to systematically study the possible origins of the giant dielectric responses in TiO_2_ ceramics codoped with +2/+5 dopants, which had never before been reported. Thus, the objective of this work was to systematically study the effects of EPDD, IBLC, and SBLC on the giant dielectric properties of Mg^2+^/Ta^5+^ codoped TiO_2_ ceramic to seek the primary origin of the giant dielectric response in that ceramic.

In this work, the Mg^2+^/Ta^5+^ codoped TiO_2_ ceramic was prepared using a solid-state reaction (SSR) method. The phase structure and microstructure of the Mg^2+^/Ta^5+^ codoped TiO_2_ ceramic were characterized. The dielectric properties of as-sintered and polished Mg^2+^/Ta^5+^ codoped TiO_2_ ceramics were investigated. A high ε′ with a low tanδ was achieved in the as-sintered Mg^2+^/Ta^5+^ codoped TiO_2_ ceramic. After removing the outer surface layers, the dielectric properties changed significantly. The dielectric properties of the polished ceramic could be recovered to the initial value by the annealing process. It was found that the temperature stability of the ε′ could be tuned by suppressing the SBLC effect. Furthermore, a broadband dielectric spectroscopy was performed to explain all possible origins of the giant dielectric phenomena in the Mg^2+^/Ta^5+^ codoped TiO_2_ ceramics.

## 2. Results and Discussion

**Figure 1:**
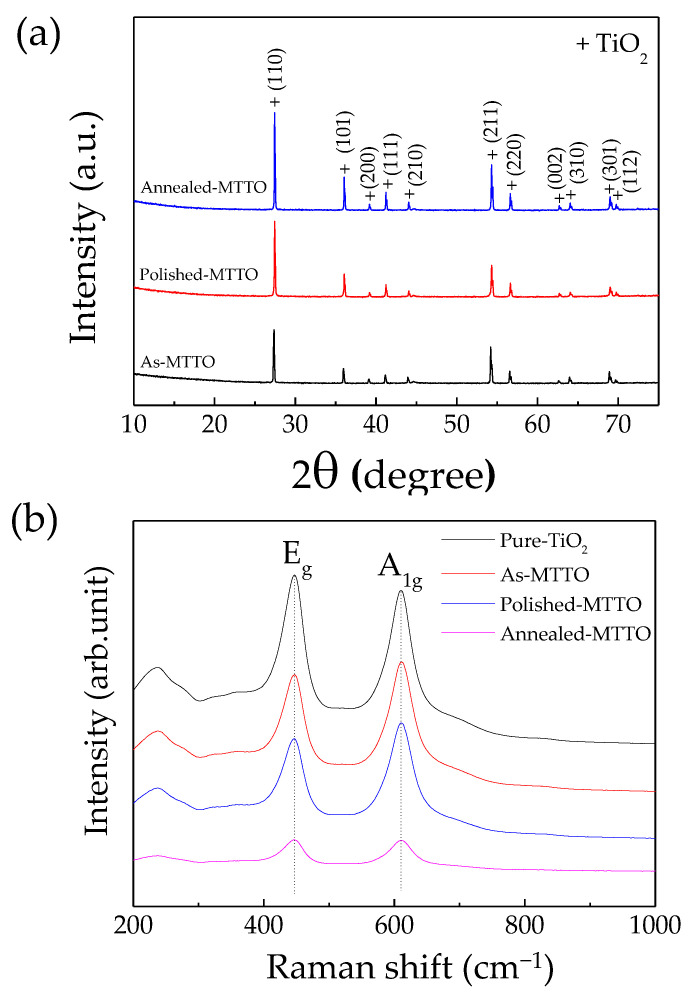
(**a**) XRD patterns of the as-MTTO, polished MTTO, and annealed MTTO ceramics sintered at 1400 °C for 2 h. (**b**) Raman spectra of the pure TiO_2_, as-MTTO, polished MTTO, and annealed MTTO ceramics.

The XRD patterns of the as-MTTO, polished MTTO, and annealed MTTO ceramics are shown in Figure 1a. A single phase of rutile TiO_2_ was obtained in all ceramic samples (JCPDS #21-1276). No impurity was detected, indicating the substitution of TiO_2_ by Mg^2+^ and Ta^5+^ due to the small mismatch of the ionic radii between Ta^5+^ (*r_6_* = 64 pm), Mg^2+^ (*r_6_* = 72 pm), and Ti^4+^ (*r_6_* = 60.5 pm). This result was similar to other acceptors/donors codoped TiO_2_ systems reported in the literature [18,21,24]. There was no segregation of any additional phase on the outer surface and in the inner core of the ceramic samples. Furthermore, the annealing process (800 °C for 30 min) did not result in the segregation of a new phase. Thus, it was expected that only defects on the outer surface would change. The lattice parameters (*a* and *c* values) of all the ceramics are shown in Table 1. The *a* or *c* value for all the ceramic samples was nearly the same owing to the same codoping concentration. Nevertheless, the *a* and *c* values of all the ceramic samples were still larger than those pf the undoped rutile TiO_2_, confirming that the Mg^2+^ and Ta^5+^ can completely replace Ti^4+^ ions in the crystal lattice [34].

Due to the crystallite size, modification of the cell parameters can also affect the Raman intensity and peak position. Thus, the Raman spectra spectroscopy of the pure TiO_2_, as-MTTO, polished MTTO, and annealed MTTO were needed, displayed in Figure 1b. The pure TiO_2_ showed the highest intensity of Raman peak, indicating its highest crystallinity [35]. The Raman peak intensity of TiO_2_ decreased with codoping with Mg^2+^/Ta^5+^. The codoping enhanced the distortion of the host lattice, reducing its crystallinity. For this reason, the annealing process was modifying the defect equilibrium that may reduce the crystallinity on the surface. The Raman active modes (i.e., *B*_1g_, *E*_g_, and *A*_1g_) were observed. The *A*_1g_ peak corresponded to the symmetric stretching of the O–Ti–O bonds in [110] plane, while the *E*_g_ peak was due to oxygen atom liberation along with the *c*-axis being out of phase [17,36,37,38]. The peak positions of the *E*_g_ and *A*_1g_ modes are summarized in Table 1. It is worth noting that the peak positions of the *E*_g_ and *A*_1g_ modes for the as-MTTO, polished MTTO, and annealed MTTO ceramics did not change due to the same codoping concentration. Generally, the existence of oxygen vacancies in MTTO ceramics has been attributed to the Mg^2+^ doping ions according to the following Equation [17]:

(1) $$MgO\overset{TiO_{2}}{\to }Mgi_{Ti}^{\prime \prime }+V_{o}^{••}+O_{o}$$

The concentrations of defects, especially for oxygen vacancies, on the surfaces of the as-MTTO, polished MTTO, and annealed MTTO ceramic samples were slightly different. However, the slight differences in defects may have had a pronounced effect on the dielectric properties of the MTTO ceramics samples.

**Figure 2:**
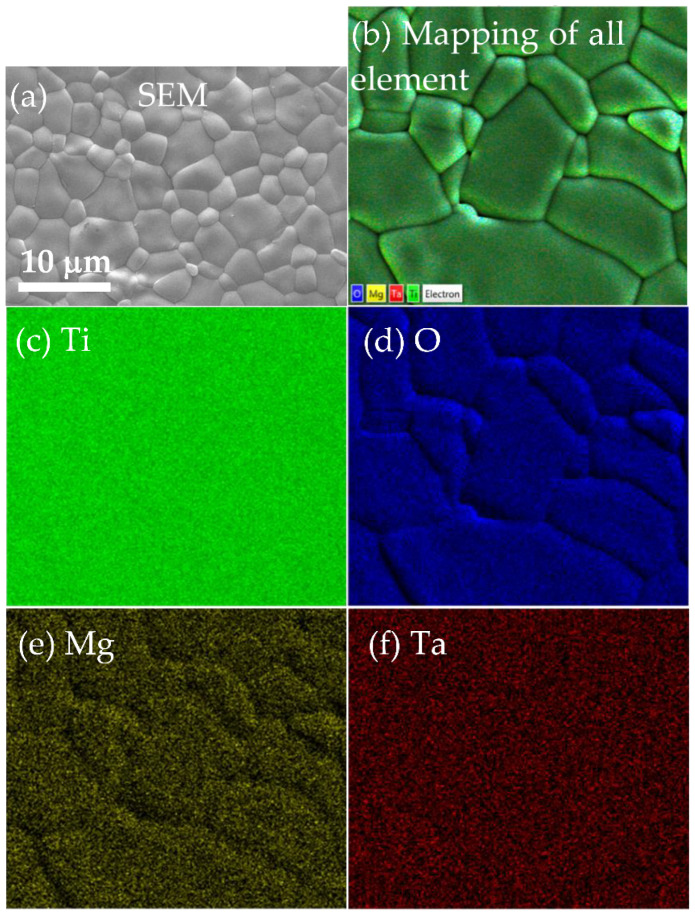
SEM image of an as-MTTO ceramic sample (**a**) and FE-SEM mapping images of all elements (**b**), Ti (**c)**, O (**d)**, Mg (**e**), and Ta (**f**).

Figure 2a shows the dense microstructure of the as-MTTO ceramic sample, with an average grain size of around 3.9 ± 1.3 µm. Furthermore, the SEM mapping image of all elements in the as-MTTO ceramic sample is shown in Figure 2b. The element mapping images of the Ti, O, Mg, and Ta are shown in Figure 2c–f, respectively. There was no segregation of any elements in a specific region.

The dielectric properties as a function of the frequencies of the as-MTTO, polished MTTO, and annealed MTTO ceramics were studied. Usually, the ε′ value of the undoped TiO_2_ ceramic is about 250 due to a lattice vibration or ionic polarization [39]. The ε′ value of the Ta^5+^ (or Nb^5+^) single doped TiO_2_ ceramic be extremely increased to 10^4^–10^5^ in the radio frequency range, while the tanδ value can also be significantly increased [14,16,40]. The simultaneously increased ε′ and tanδ values can be explained by electron hopping [14,15,16,24,41]. The substitution of rutile TiO_2_ with Ta^5+^ can produce free electrons, according to the following Equations:

(2) $$\;2TiO_{2}+Ta_{2}O_{5}\overset{4TiO_{2}}{\to }2Ta_{Ti}^{•}+2Ti_{Ti}^{\prime }+8O_{0}+0.5O_{2}$$

(3) $$Ti^{4+}+e\to Ti^{3+}$$

**Figure 3:**
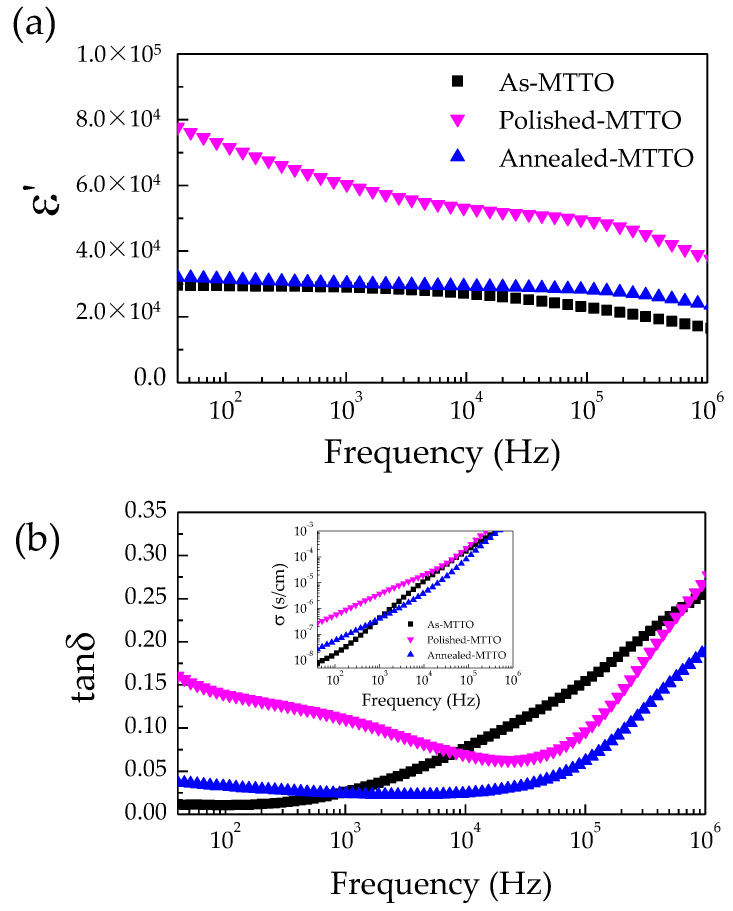
Frequency dependence of (**a**) ε′ and (**b**) tanδ at RT in as-MTTO, polished MTTO, and annealed MTTO ceramics. The inset of (**b**) shows the conductivity at RT in the frequency range of 40–10^6^ Hz.

Nevertheless, the substitution of Ta^5+^ (or Nb^5+^) doped TiO_2_ with acceptor dopants (e.g., Ag^+^, Zn^2+^, or In^3+^) can cause a significant decrease in the tanδ value [14,16,20,23,40]. The reduced tanδ value has been explained by many factors such as defect clusters [14], insulating grain boundaries [20,42], and insulative outer surface layers [16,28,29,43]. It was reported that a high ε′ and low tanδ can be obtained in as-sintered In^3+^/Nb^5+^ codoped TiO_2_ ceramics or annealed In^3+^/Nb^5+^ codoped TiO_2_ ceramics [28,29,42]. The tanδ value can be significantly increased after removing the outer surface layer [28,42]. As illustrated in Figure 3a,b, a high ε′ (>10^4^) over the measured frequency range can be obtained in the as-MTTO ceramic, while a low tanδ (<0.05) was achieved in the frequency range below 2 kHz. After removing the outer surface layer, the ε′ of the polished MTTO ceramic was increased by a factor of two compared to that of the as-MTTO ceramic. However, the tanδ value of the polished MTTO ceramic was also increased by more than two times over the measured frequency range. Surprisingly, the ε′ value of the annealed MTTO ceramic could be returned to the initial value of the as-MTTO ceramic by annealing in air. Furthermore, a low tanδ (<0.05) could be extended to a higher frequency range of 10^5^ Hz compared to that of the as-MTTO ceramic. This observation indicates that the outer surface layer had a remarkable influence on the overall dielectric properties of the MTTO ceramics, which was similar to that observed in other codoped TiO_2_ systems such as Ga^3+^/Ta^5+^ [16], In^3+^/Nb^5+^ [28], and Al^3+^/Nb^5+^ [17]. The ε′ values at 1 kHz of the as-MTTO, polished MTTO, and annealed MTTO ceramics were 28,970, 60,221, and 30,249, respectively, while the tanδ values at 1 kHz were 0.026, 0.110, and 0.024, respectively. The dielectric properties of the polished MTTO ceramic are comparable those +2/+5 codoped TiO_2_ systems, as summarized in Table 2.

After removing the outer surface layer, the tanδ and associated low frequency conductivity of the polished MTTO ceramic (inset of Figure 3b) increased significantly, indicating the insulating nature of the outer surface layer. After removing the insulating surface layer, the outer surface of the polished MTTO ceramic became a semiconductor. Generally, the Schottky barrier height (SBH) can be created at the interface between the electrode and semiconducting core in accordance with the Schottky–Mott theory [44]. The SBH is dependent on the different work functions between the electron affinity of the semiconductor and that of the metal electrode. Therefore, the highest ε′ value of the polished MTTO ceramic has been attributed to the artificial interfacial polarization at the sample–electrode interface, which is associated with DC conduction. However, this is usually accompanied by an increase in tanδ. Note that the SBH could not be created on the surface of the as-MTTO ceramic due to its insulating outer surface skin. The dielectric properties of the annealed MTTO ceramic were almost entirely returned to the initial properties of the as-MTTO ceramic. These results indicate that the formation of the SBH was inhibited in the annealed MTTO ceramic due to its insulating outer surface layer, just like the as-MTTO ceramic. Typically, the annealing process can fill oxygen vacancies on the surfaces and/or along the grain boundaries, converting the semiconducting surface to an insulating surface, according to Equation (4) [45].

(4) $$\frac{1}{2}O_{2}+V_{o}^{••}+2e^{\prime }\to O_{O}^{x}$$

**Figure 4:**
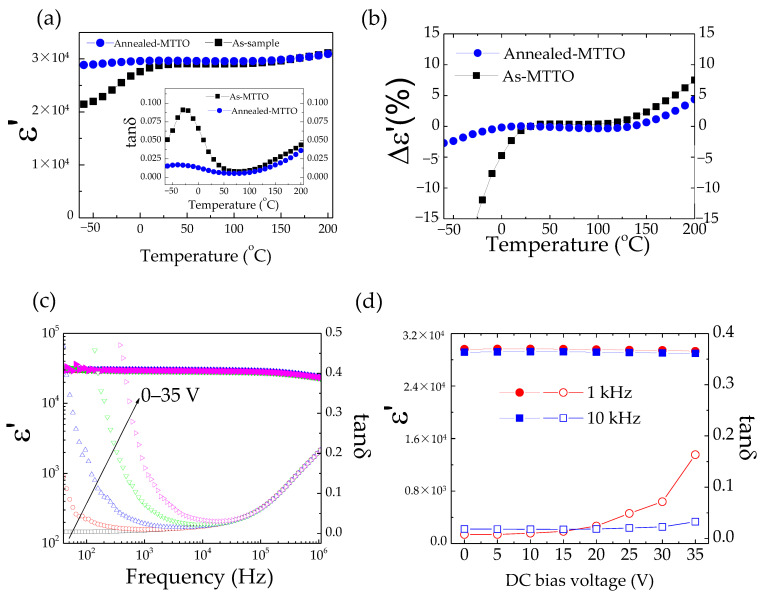
(**a**) Temperature dependence of ε′ for as-MTTO and annealed MTTO ceramics at 1 kHz; the inset shows the temperature dependence of the tanδ of the as-MTTO and annealed MTTO ceramics at 1 kHz. (**b**) Temperature coefficient of ε′ at 1 kHz for as-MTTO and annealed MTTO ceramics. (**c**) Frequency dependence of ε′ at RT under different DC voltage biases for the annealed MTTO ceramic. (**d**) DC bias voltage dependence of ε′ at 10^3^ and 10^4^ Hz for the annealed MTTO ceramic.

Notably, it was also observed that the dielectric properties of the annealed MTTO ceramic were better than those of the as-MTTO ceramic. The ε′ of the annealed MTTO ceramic was more stable in frequency than that of the as-MTTO ceramic, while a low tanδ (<0.05) value was extended to a higher frequency range. The temperature dependence of the dielectric properties of the as-MTTO and annealed MTTO ceramics was studied. As illustrated in Figure 4a, the ε′ values of as-MTTO and annealed MTTO ceramics were nearly the same in the temperature range of 20–210 °C. However, a significant difference in ε′ values was observed below 20 °C. A step-like decrease in the ε′ was observed in the as-MTTO ceramic, which was accompanied by the appearance of tanδ peak, as shown in the inset of Figure 4a. This result may indicate the different electrical properties of the surface layers and/or grain boundaries between the as-MTTO and annealed MTTO ceramics [46]. After the annealing process, the ε′ of the annealed MTTO ceramic shows a plateau over the temperature range of −60–200 °C, while the step-like decrease in the ε′ disappeared. Hence, the annealing process could improve the temperature stability of the ε′ in a low temperature range. As displayed in Figure 4b, the temperature coefficients’ (Δε′(*T*)/ε′_*RT*_) values at different temperatures indicate the fabulous performance of the dielectric properties of the as-MTTO and annealed MTTO ceramics. Interestingly, the temperature coefficient of the annealed MTTO ceramic was in the range of ±15% between −60 and 200 °C, while the temperature coefficient of the as-MTTO ceramic was very large in a low temperature range. Furthermore, at 1 kHz and 200 °C, the Δε′(200 °C)/ε′_*RT*_ of the annealed MTTO ceramic was as low as 4.38%. The properties were much better than those of CCTO [1], CuO [44], NiO-based oxides [9], as well as other acceptor/donor codoped TiO_2_ [16,21,24].

**Table 2:** ε′ and tanδ at 1 kHz and room temperature of +2/+5 ions codoped TiO_2_ ceramics.

Codoped TiO_2_	ε′	tanδ	Reference
(Zn_1/3_Nb_2/3_)_0.05_Ti_0.95_O_2_	~30,000	~0.05	[19]
(Ca_1/3_Nb_2/3_)_0.01_Ti_0.99_O_2_	130,500	0.19	[47]
(Mg_1/3_Tab_2/3_)_0.005_Ti_0.995_O_2_	>7000	0.002	[24]
(Cu_1/3_Ta_2/3_)_0.2_Ti_0.8_O_2_	65,314	>0.1	[48]
(Sr_1/3_Ta_2/3_)_0.05_Ti_0.95_O_2_	186,000	0.15	[41]
(Mg_1/3_Ta_2/3_)_0.01_Ti_0.99_O_2_	30,249	0.024	This work

To further explore the dielectric performance of the annealed MTTO ceramic, the effects of DC bias on the ε′ and tanδ values were measured in the range of 0–35 V, shown in Figure 4c. The ε′ was independent of the DC bias voltage. At the same time, tanδ was significantly increased in a low-frequency range. Figure 4d shows the ε′ and tanδ values at the selected frequencies of 1 kHz and 10 kHz. The ε′ value was nearly independent of the DC voltage bias. The tanδ at 10 kHz was also only slightly dependent on the DC bias voltage, whereas the tanδ at 1 kHz tended to change with DC bias at ≥ 25 V. This may have been caused by the release of trapped electrons from internal interfaces caused by an applied DC bias—i.e., the interface between semiconducting grains and insulating grain boundary and the interface between the semiconducting core and insulative outer surface layer. Generally, the SBH can be created at the interface between *n*-type grains and interfaces between a resistive outer surface layer and a semiconducting inner core [44]. Under an applied DC bias, the Schottky barrier is asymmetric, meaning that the depletion width in the forward direction decreases [49]. This results in a decreased SBH in this direction, leading to an increased conductivity and associated tanδ value [16,49]. The ε′ and tanδ in a high range (>10^4^ Hz) was not affected by the DC bias below 35 V. This indicated that DC bias did not affect the primary source(s) of the polarization that give(s) rise to the high dielectric performance of annealed MTTO ceramic. The giant dielectric properties were enhanced by various factors (i.e., electron-pin defect dipoles, grain boundary effect, and insulative surface skin).

**Figure 5:**
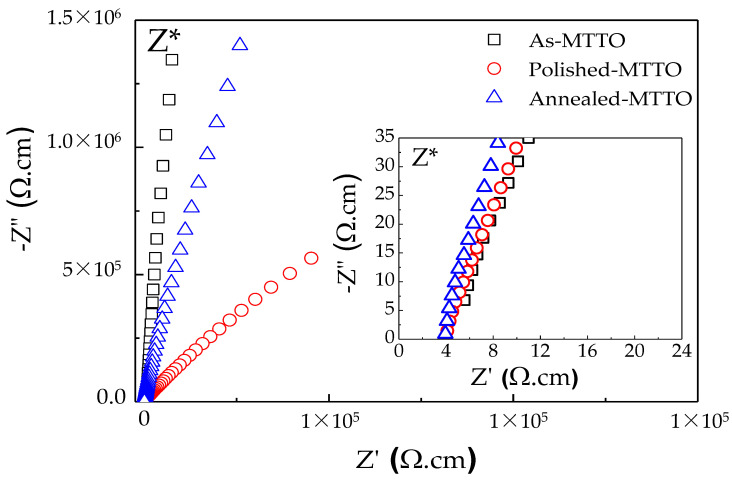
Impedance complex plane plots (Z*) for the as-MTTO, polished MTTO, and annealed MTTO ceramics at RT. The inset shows Z* plots of the high frequency data near the origin.

Figure 5 demonstrates the impedance complex plane plots, i.e., Z* = Z′−*j*Z″ (where, $j=\sqrt{-1}$. Z′ and Z″ are the real and imaginary parts of Z*, respectively), for as-MTTO, polished MTTO, and annealed MTTO ceramics at RT. The inset shows Z* plots of the high frequency data near the origin. Usually, the large semicircular arc of Z* plots is due to the electrical response of the insulating grain boundaries and/or surface layers, while the nonzero intercept is usually described by the semiconducting grains [25,28,40,43,49]. The total resistance of the insulating parts and grain resistance can be calculated by the diameter of the large arc and nonzero intercept on the Z′ axis, respectively. As clearly seen in Figure 5, although an entire arc of each ceramic cannot be observed, it can be estimated that the total resistance of the insulating part of the polished MTTO ceramic was reduced and lower than those of the as-MTTO and annealed MTTO ceramics. This result clearly indicates that the outer surface layer of the MTTO ceramics was an insulator. Nevertheless, the diameter of the large arc of the polished MTTO ceramic was still too large, indicating the primary electrical response of grain boundary. After removing the insulative outer surface layer, the conductivity and associated tanδ value increased. It was also observed that the grain resistances of all the MTTO ceramics were nearly the same. Removing the insulative outer surface layer or annealing the MTTO ceramic did not change the electrical properties of the semiconducting grains. According to impedance spectroscopy, the giant dielectric properties of the MTTO ceramics can likely be attributed to interfacial polarization at the internal interfaces. However, the effect of the EPDDs cannot be ignored.

Although the origins of the giant dielectric properties of the MTTO ceramic systems may be caused by many factors, the giant dielectric properties of the MTTO ceramics are fascinating due to a high ε′ and low tanδ over a wide temperature range. Furthermore, the ε′ value was slightly dependent on the temperature ranging from −60 to 200 °C, meeting the X9R standard capacitor. This work provides comprehensive guidelines for extending the temperature stability within a low temperature range.

## 3. Experimental Details

### 3.1. Sample Preparation

When the codoping concentration increases, a self-charge compensation between 2Ta^5+^-Mg^2+^ may exist. In this case, both the EPDD or interracial polarization (IBLC and/or SBLC) cannot occur. To avoid this possible self-charge compensation, the codoping concentration was fixed at 1.0%. Thus, the (Mg_1/3_Ta_2/3_)_0.01_Ti_0.99_O_2_ (MTTO) was prepared using the SSR method. First, TiO_2_ (Sigma-Aldrich, St Louis, MO, USA, 99.9% purity), MgO (Sigma-Aldrich, ≥99.99% purity), and Ta_2_O_5_ (Sigma-Aldrich, 99.99% purity) were mixed by wet ball milling in ethanol for 24 h using ZrO_2_ balls (≈2.0 mm in diameter). Second, the mixed slurry was heated at ~80–100 °C to evaporate the ethanol until a mixed dried powder was achieved. Third, the dried powder was carefully ground and pressed into pellets by uniaxial compression at ≈180 MPa. Finally, the pellets were sintered at 1400 °C for 2 h in air using a 3 °C/min heating rate followed by natural furnace cooling to room temperature (RT).

### 3.2. Dielectric and Electrical Measurements

In this work, the effects of insulative outer surface layers on the dielectric and electrical properties of the MTTO ceramic were studied. First, both sides of the surfaces of the as-sintered sample (as-MTTO) were pasted with silver paint and fired at 600 °C for 30 min in the air in order to make electrodes. The dielectric properties of the as-MTTO ceramic were measured under an AC oscillation voltage of 0.5 V using a KEYSIGHT E4990A Impedance Analyzer (Santa Rosa, CA, USA) over the frequency and temperature ranges of 40–10^7^ Hz and −60–210 °C, respectively. Each measured temperature was kept constant with a precision of ±0.1 °C.

Second, the initial electrodes and both sides of the outer surfaces of the as-MTTO ceramic were removed by polishing with SiC paper. The thickness surface layer of each side that was removed was 0.13 mm. The polished MTTO sample was cleaned by an ultrasonic sonicator for 30 min in deionized water and dried at 80–100 °C in a furnace. The silver paint was pasted on both sides of the polished surface and then fired at 600 °C for 30 min (polished MTTO). The dielectric and electrical properties of the polished MTTO sample were studied.

Third, the electrodes on the polished MTTO sample were removed by polishing. The skin surfaces of the polished MTTO sample were gently polished and cleaned by an ultrasonic sonicator for 30 min in deionized water. The sample was annealed in the air at 800 °C for 30 min and subsequently pasted with silver paint and fired at 600 °C for 30 min (annealed MTTO). The dielectric and electrical properties of the annealed MTTO sample were measured. In addition, the dielectric and electrical measurements were performed under different DC bias levels (0–35 V).

### 3.3. Characterizations

To explain the effect of the outer surface layer on the dielectric and electrical properties of MTTO ceramics, the as-MTTO, polished MTTO, and annealed MTTO samples were systematically characterized as follows. The phase crystalline structure and microstructure of a sintered ceramic were characterized by an X-ray diffraction (PANalytical, EMPYREAN) (Shanghai, China) and scanning electron microscopy (SEM) (SEC, SNE4500M) (Suwon, Korea), respectively. The SEM mapping technique was performed using a focus ion beam field emission scanning electron microscopy (FIB-SEM, FEI Helios NanoLab G3 CX) (Dreieich, Germany) with energy dispersive X-ray analysis (EDS) to reveal elemental distribution in the MTTO ceramic. Raman spectra were collected using a UV–vis Raman System (Bruker, SENTERRA II) (Ettlingen, Germany) with an excitation wavelength of 532 nm.

## 4. Conclusions

The MTTO ceramics were prepared by the SSR method. The as-MTTO ceramic could exhibit a giant ε′ value and lowδ. By removing the outer surface layer, the ε′ and tanδ increased significantly. Also, those values could be returned to the initial value of the as-sintered ceramic after annealing in air. Excellent dielectric performance properties were achieved in the annealed sample, showing ε′ ≈ 30,249 with tanδ ≈ 0.024. Interestingly, the ε′ value at 1 kHz slightly changed from −2.7% to 4.38% in the temperature range of −60 to 200 °C, while the loss tangent was very low (~0.015–0.036 at 10^3^ Hz) in this temperature range. The excellent dielectric properties might be enhanced by the insulating grain boundaries and insulative outer surface layer. However, the EPDD effect cannot be ignored.

## Figures and Tables

**Table 1:** Lattice parameters and Raman shift of pure TiO_2_ and as-, polished and annealed MTTO ceramics.

Sample	Lattice Parameters (Å)		Raman Shift (cm^−1^)	
	*a*	*c*	*E_g_*	*A*_1*g*_
Pure TiO_2_	-	-	447	610.0
As-MTTO	4.597	2.963	447	611.5
Polished MTTO	4.597	2.963	446.5	610.5
Annealed MTTO	4.597	2.963	446.5	610.5

## Data Availability

The data presented in this study are available in article.

## Sample Availability

Samples of the compounds are not available from the authors.
